# Breast Cancer Detection Using Infrared Thermal Imaging and a Deep Learning Model

**DOI:** 10.3390/s18092799

**Published:** 2018-08-25

**Authors:** Sebastien Jean Mambou, Petra Maresova, Ondrej Krejcar, Ali Selamat, Kamil Kuca

**Affiliations:** 1 Center for Basic and Applied Research, Faculty of Informatics and Management, University of Hradec Kralove, Rokitanskeho 62, Hradec Kralove 500 03, Czech Republic; jean.mambou@uhk.cz (S.J.M.); petra.maresova@uhk.cz (P.M.); aselamat@utm.my (A.S.); kamil.kuca@uhk.cz (K.K.); 2 Faculty of Computing, Universiti Teknologi Malaysia, Johor 81310, Malaysia

**Keywords:** breast, cancer, detection, visual techniques, neural network, SVM, deep learning, DNN, RNN

## Abstract

Women’s breasts are susceptible to developing cancer; this is supported by a recent study from 2016 showing that 2.8 million women worldwide had already been diagnosed with breast cancer that year. The medical care of a patient with breast cancer is costly and, given the cost and value of the preservation of the health of the citizen, the prevention of breast cancer has become a priority in public health. Over the past 20 years several techniques have been proposed for this purpose, such as mammography, which is frequently used for breast cancer diagnosis. However, false positives of mammography can occur in which the patient is diagnosed positive by another technique. Additionally, the potential side effects of using mammography may encourage patients and physicians to look for other diagnostic techniques. Our review of the literature first explored infrared digital imaging, which assumes that a basic thermal comparison between a healthy breast and a breast with cancer always shows an increase in thermal activity in the precancerous tissues and the areas surrounding developing breast cancer. Furthermore, through our research, we realized that a Computer-Aided Diagnostic (CAD) undertaken through infrared image processing could not be achieved without a model such as the well-known hemispheric model. The novel contribution of this paper is the production of a comparative study of several breast cancer detection techniques using powerful computer vision techniques and deep learning models.

## 1. Introduction

The human body naturally manages the creation, growth, and death of the cells in its tissues. Once this process starts to work abnormally, and the cells are not dying at the rate they should, we see an increase in the ratio of cell growth to cell death, which is a direct cause of cancer. Breast cancer occurs when cells in the breast divide and grow without reasonable control. It is a well-known disease around the world; in the USA, one in eight women will be diagnosed with breast cancer in her lifetime, and more than 40,000 is dying every year in USA [1]. We can reduce this number using early detection techniques, awareness campaigns, and better diagnostics, as well as by improving treatment options. In this paper, we will first explore infrared digital imaging, which assumes that a basic thermal comparison between a healthy breast and a breast with cancer always shows an increase in thermal activity in the precancerous tissues and areas surrounding developing breast cancer. This is due to the metabolic activity and vascular circulation occurring in and around cancerous cells. Over the last 20 years several techniques have been proposed to better manage breast cancer, such as mammography, which is one of the most common techniques for the diagnosis of breast cancer [2,3]. Our goals are:

- To give a clear picture of breast cancer detection using infrared images.
- To propose a model powerful enough to help in the early detection of breast cancer.

## 2. Previous Techniques and Comments

**Figure 1:**
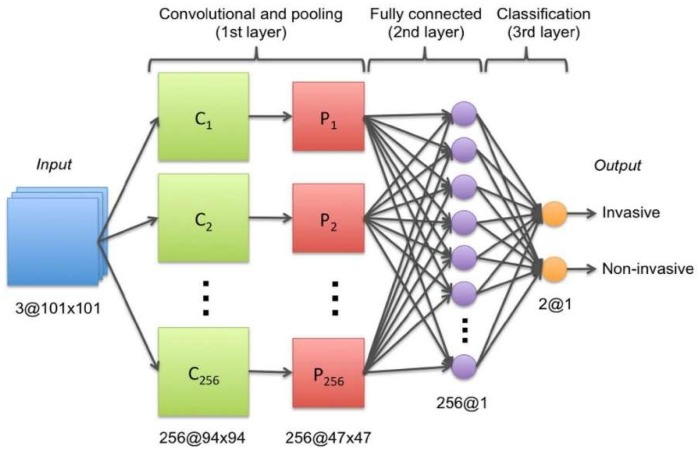
ConvNet architecture used to determine the state of the cancer (invasive or non-invasive) [5].

Based on several articles from 2002–2010, the authors of previous related research Kosus et al., 2010 [4] undertook a review that illustrates the limitations of screen-film mammography (SFM). For example, a large number of false positives occur with this technique, with a rate between 4% and 34%. There is a considerable amount of interest in image processing through a network of neurons. To be more precise, several researchers [5,6] have explored the detection and assessment of severity of cancer in this manner. Figure 1 shows how, thanks to ConvNet, we can classify cancer as invasive or non-invasive. In addition, some authors [7,8,9,10,11,12,13] have demonstrated the power of the neural network model (NN) and its variants (Recurrent Neural Network (RNN), Deep Neural Network (DNN), etc.). They also stressed the importance of solving the problem of cancer of the breast through innovative techniques in computer science.

The authors in [3] presented an overview of the recent state-of-the-art deep learning-based Computer Aid Design (CAD) systems developed for mammography and breast histopathology images. They also describe the relationship between mammography and histopathology phenotypes, which takes biological aspects into account. They propose a computer-based breast cancer modeling approach which develops a mapping of features/phenotypes between mammographic abnormalities and their histopathological representation. In the same way, the authors in [14] have investigated the use of Local Quinary Patterns (LQP) for breast density classification in mammograms on various neighbourhood topologies. They took a multiresolution and multi-orientation approach, studied the effects of multiple neighbourhood topologies and selected dominant patterns to maximise texture information. Nevertheless, they used a Support Vector Machine classifier (SVM) to perform the classification of the data obtained.

Despite the preference for mammography for the past several decades, the need for new techniques to overcome the limitations of mammography as a technique has emerged. In the same way, research has presented near-infrared fluorescence (NIRF) as an essential element in the cancer diagnostic process [15], as well as in the continuous observation of the disease and the treatment of the disease. It is essential that the image processing has a powerful NIRF light signal, so that the image taken contains a lot of information that is very close to the actual state of the breast. As we know, the sooner the tumor is found, and the sooner treatment is consequently begun, the better the chances of success. Other researchers have discussed the difficulty of obtaining tumor parameters such as the metabolic heat, tumor depth, and diameter of the thermogram [16]. Another article [17] mentions the limitations of computed tomography (CT) and magnetic resonance imaging (MRI), which have low sensitivity for sub-centimeter lesions because of their limited spatial resolution. Some research has shown further negative points of successive mammography tests occurring over a period of 10 years [18]. According to the study performed by Kandlikar et al., 2017 [18], the false positive diagnosis rate for women after having a mammogram each year for 10 years is 49.1%. Another study showed that when women were advised to do an NLS (Sentinel Lymph Node) biopsy, this reduced the risk of progression of the disease (breast cancer) [19]; moreover, other authors Kontos et al., 2011 [20] have advised against taking breast cancer thermography results as sufficient information for decision-making.

### 2.1. Related Studies

**Doubt in infrared techniques:** Kontos et al., 2011 [20] illustrated the limitations of the thermographic image as an essential tool in technical decision-making because of its high number of false positives, observed by performing, for example, a test on 126 breasts of 63 patients (58 women and 5 men). The average age of the patients was 47.6 years (range 26–82 years). After treatment of the thermal image, the cancerous lesions were diagnosed in 20 breasts, and there were no bilateral cancers. Overall, the study concluded that thermal diagnosis was not sufficient for the initial evaluation of symptomatic patients.

**Infrared thermal imaging:** Kosus et al., 2010 [4] discussed pre-digital mammography (FFDM) and digital infrared thermal imaging (DITI) as imaging modalities that could overcome the limitations of mammography. Considering its ability to selectively optimize contrast in areas of dense parenchyma, we observed a significantly better performance of digital mammography compared to screen-film mammography in younger women with dense breasts. On the other hand, the use of infrared digital imaging is based on the principle that metabolic activity and vascular circulation in precancerous tissues and areas surrounding developing cancer are almost always higher than in normal tissues. The results of the thermography can be correct 8–10 years before a mammogram can detect a mass in the body of the patient. In addition to this, a shortage of qualified radiologists has caused an urgent need for the development of computer technologies like Computerized Thermal Imaging (CTI), which analyzes and interprets a series of infrared images using algorithms that correlate infrared data regarding the breast being examined with infrared models associated with benign or malignant breast tissue. It does not require any physical contact, and there are no liquids to drink. Furthermore, it does not use ionizing radiation, making it highly preferable in diagnostic procedures for pregnant women and younger women. In 1982, the United States Food and Drug Administration (FDA) approved thermography as a supplement to mammography to help detect breast cancer. However, it is essential to note that CTI is more valuable in clinical use due to its high negative predictor value, compared to its positive predictive value which does not have much clinical utility. CTI is considered here as a parallel process of cancer detection—this article does not encourage the use of the thermal image alone for decision-making, but shows the importance of CTI in several cases where the use of mammography can be risky for the patient [21].

**Figure 2:**
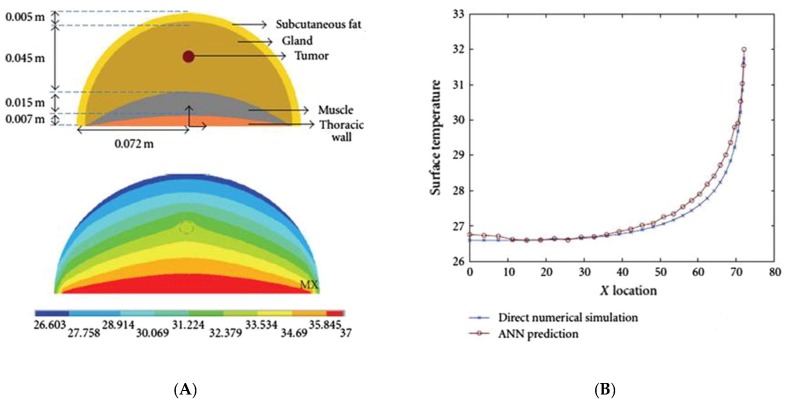
Illustration of thermal diffusion in a conventional breast representation [22]. (**A**) The computational domain as a hemisphere semi-spherical. (**B**) Numerical simulation and surface temperatures.

**Figure 3:**
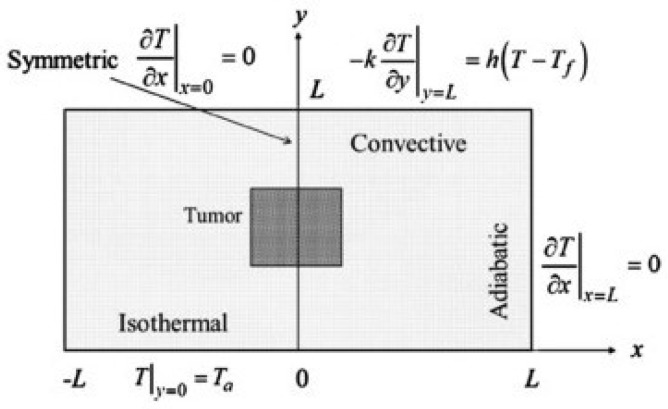
The rectangular area that provided the first glimpse of predictive models that relate surface temperature to tumor size and location [23].

**Figure 4:**
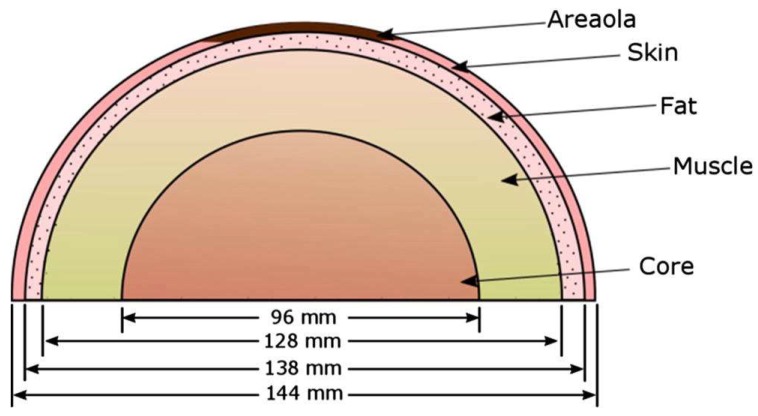
Hemispherical domain with non-concentric layers, which is a prevalent model because of its ability to reproduce the surface temperature [22,24].

The research by Boogerd et al., 2017 [17] discussed thermal diagnosis, which is still improving every year. Several thermal diagnoses have shown that breast cancer patients with abnormal thermograms have fast-growing tumors. For better test results, a protocol must be followed before a patient undergoes an examination—for the test procedure, for the environment during the study, and for the post-treatment of the obtained thermograms. The thermal diagnosis can be further optimized using the Artificial Neural Network (ANN), for which many images both with cancer and without breast cancer must be provided to feed the input layer, and are then processed in the hidden layers. The output of the last hidden layer serves as input to the neurons in the output layer, and a decision is made following this. Combining ANNs, genetic algorithms, and computer simulations to relate skin surface temperature to depth, diameter, and heat generation, considering the computational domain as a hemisphere semi-spherical (Figure 2a), may offer further improvements in diagnosis. Additionally, it is worth noting that the ANN has shown good agreement with numerical simulations and surface temperatures (Figure 2a,b). To better understand breast cancer, several geometric models have been proposed so far, including the rectangular domain (Figure 3) which provides the first overview of predictive models that relate surface temperature to the size and location of the tumor; it does not represent the actual shape of the breast however. The hemispheric computational domains shown in Figure 4 gave results more in agreement with the experimental data. The temperature distribution illustrated the effect of the different layers on the surface temperature. However, the hemispherical domain with non-concentric layers is a prevalent model because of its ability to reproduce surface temperatures that are in close agreement with the experimental data. One inaccuracy of this model is its symmetric temperature distribution, which is at odds with other experimental observations made by Ng (E.Y.K. Ng, E.C. Kee). In areas measured with the actual breast shape in mind, it appeared that gravity deformations create an asymmetric temperature distribution with a warmer region on the upper quadrant of the breast and a colder region in the lower quadrant, however it remains to be seen whether the asymmetric temperatures of the distribution are due to high vascularity or mechanical deformity of the breast. As an effect of the tumor, the article illustrates the correlation between the depth of the tumor and the temperature at the surface. A ratio of 1:3 (diameter to depth) sets the limit for the possible variations of the temperature at the surface. As the temperature is known on the surface of the breast, it was necessary to develop a model that can help to solve the enigmatic question of the depth and diameter of the tumor, as well as its location. For this reason, a well-known model was introduced, known as Everse modeling [21].

In our context of breast tumor detection, the breast surface temperature is the solution of the natural heat transfer equation, so it is sufficient to determine the parameter value of this equation. An application of the inverse modeling method, however, requires a model for the breast. Moreover, in our context, the natural heat transfer equation is solved for a set of initial values of the physical, thermal properties of breast tissues.

**Figure 5:**
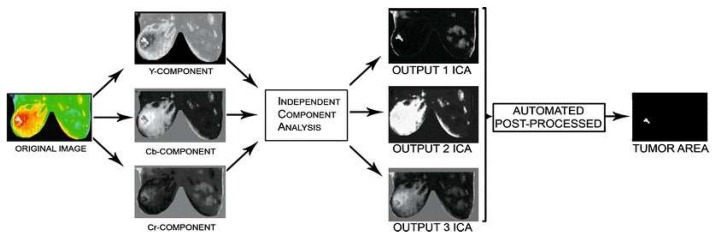
The overview of the ICA process combined with automated post-processing, used to detect a tumor in the breast more effectively [28].

**Figure 6:**
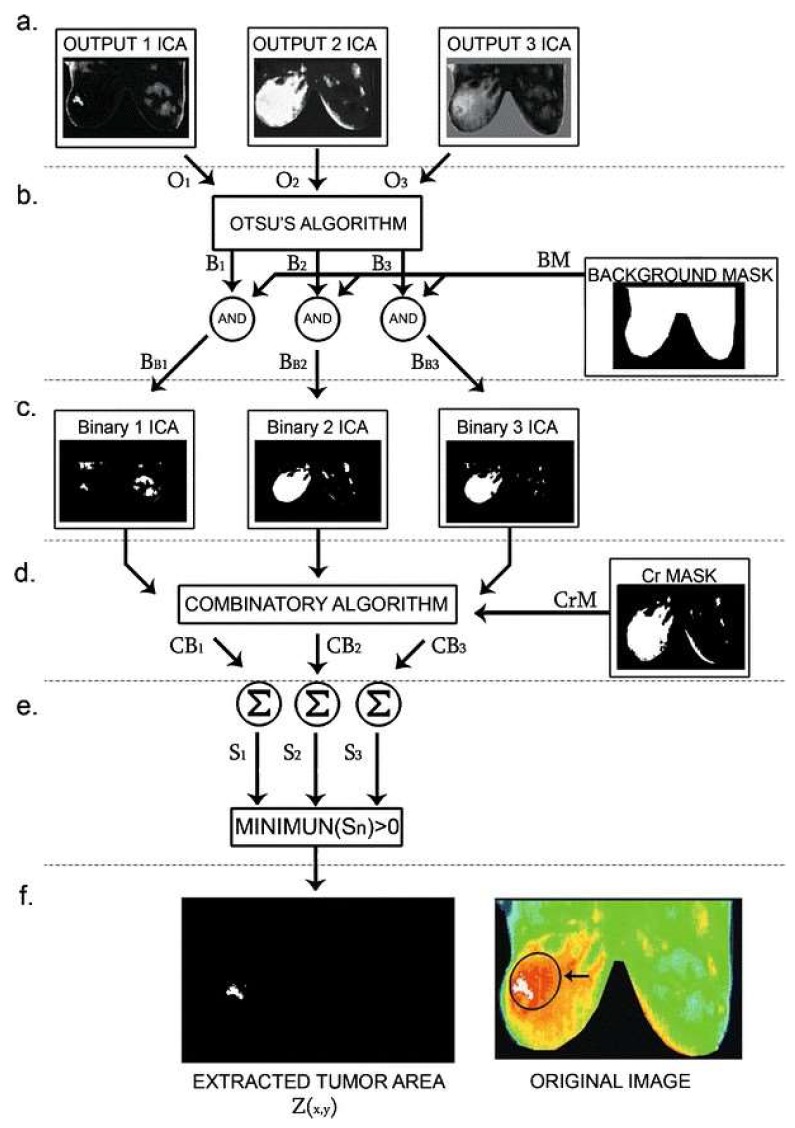
The process of extraction of the tumor area [28].

We also need techniques such as the gradient descent method, Levenberg Marquardt algorithm, or genetic algorithms to estimate the value of thermal physical properties. However, the temperature cannot be measured on the breast surface, and the temperature remains unchanged. On the other hand, the paper by Boquete et al., 2012 [25] develops IR radiation of the human body, which is most often of the order of 2–20 µm, and can be detected very precisely with modern infrared cameras [26,27]. These two factors must be kept in mind for success in the field of diagnosis and improvement of image processing algorithms. Similarly, Independent Component Analysis (ICA) is a subspace projection technique that projects data from an ample space to a smaller area. The ICA tumor analysis method is composed of three concatenated phases: Separation of the chrominance (*Cb*, *Cr*) and luminance (*Y*) components, the image of the image-independent components, and post-processing for the segmentation of the tumor areas. This article presents features that are inappreciable in the original image and that are associated with regions of high-risk tumors, due to the strong relationship between body temperature and an extreme tone in the original image. However, ICA must solve a problem that considers the *X* data matrix (digital image) as a linear combination of independent components (Figure 5), namely X=AS where *S* contains the independent components and the mixing matrix, and its coefficients describe only mixed source regions and can be used as extracted entities. In summary, ICA attempts to “disassemble” the data by estimating a demixing matrix, *W*, where Y=WX so that the objective of the ICA is to recover *A* and *Y* using the information contained in *X*. matrix W=powerA(−1) (the demixing matrix). The different results of the ICA will be given as an automated post-processing input which, using several internal calculations, will result in a comparison between the results (Figure 6).

**Figure 7:**
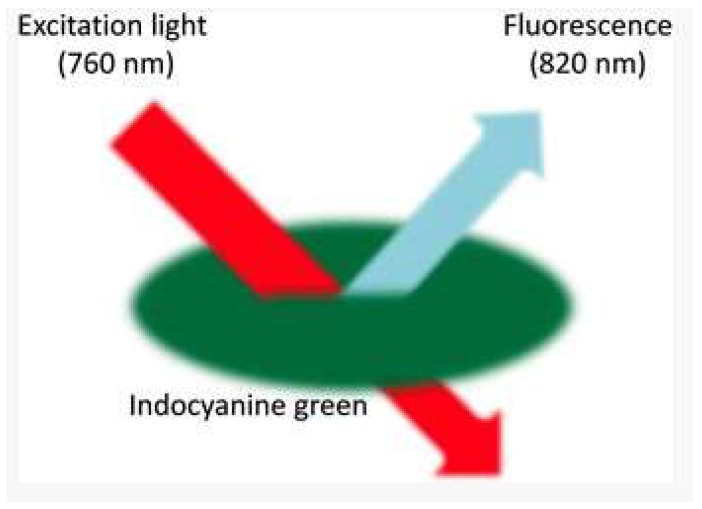
ICG fluorescence with a specific light wavelength (820 nm) in the near infrared spectrum [20].

**Near-infrared fluorescent and agent:** The article by Dongola et al., 2016 [2] introduced the concept of an agent which, in our context, represents a molecule’s composition. When absorbed, this can envelop the tumor and produce a stronger near-infrared fluorescent (NIRF) signal suitable for proper brain localization. The IR780phospholipid micelle shown in Figure 7 is ideal for this purpose because it has the ability to cross the natural barrier of the brain, and also to locate and attach to tumor cells. The research base developed by Boogerd et al [17] on fluorescence imaging (FI), as applied to liver surgery, shows FI significance during laparoscopic resections of several liver tumors. It is unlike computed tomography and magnetic resonance imaging (MRI), which have low sensitivity for sub-centimeter lesions due to their limited spatial resolution. It therefore seems that FI is suitable for surgeons to identify small superficial tumors in the liver. Surgeons need a highly sensitive, real-time intraoperative imaging modality. Additionally, the added value of fluorescence imaging during oncological liver surgery is twofold, as it can delineate liver tumors and provide real-time assessment of the resection margin, and can also identify otherwise undetectable liver tumors. The success of detecting pre- and perioperative tumors [4,15] in twenty patients has shown that the NRI fluorescent technique is an asset for surgeons. It has been found that laparoscopic ultrasound (LUS) paired with near-infrared fluorescence imaging (NIRF) can achieve a sensitivity and positive predictive value (PPV) of 100% and 70.3%, respectively. On the other hand, Tsutomu Namikawa, [19] focused on detection using the Sentinel Lymph Node (SLN). This can be defined as the first lymph node with a high risk of propagation of the primary tumor, and it is detected using an SLN sentinel biopsy to reduce the risk of progression of the disease. To perform an SLN, Indocyanine Green (ICG) is administered to the patient before the examination. ICG is a hydrophilic tricarbocyanine dye with a molecular weight of 776 Da, which rapidly binds to plasma proteins in the body. ICG becomes fluorescent with a specific light wavelength (820 nm) in the NIR spectrum (Figure 7). As this article shows, the ICG was well tolerated with no immediate or short-term complications related to its administration. NIR fluorescence imaging using ICG is not only feasible and safe for the intraoperative detection of SLN, allowing real-time observation without training, but also gives high detection rates and false negatives. One application is the HyperEye Medical System (HEMS, Mizuho Co., Ltd., Tokyo, Japan) which was developed to simultaneously detect NIR rays under room light to provide color imaging, giving the significant benefit of navigation during surgery [29].

**Diameter and depth of the tumor:** The paper by Kosus et al., 2010 [4] shows the diameter and depth of the tumor as a problem to be solved using thermal techniques, using the width at half height (FWHM) to estimate the depth of a small heat source of the isothermal distribution hot spots area.

Furthermore, because of the complex structure of breast heat transfer, the application of a vascular model requires detailed knowledge of the microvascular network. Therefore, the Penne bioheat Equation (1) was used to model the heat transfer in the breast:

(1) $$pc\frac{\delta T}{\delta t}=\nabla \cdot k\nabla T-w_{b}p_{b}C_{b}\left(T-T_{b}\right)+O_{m}$$

where *p*, *c* and *k* denote the density, specific heat, and thermal conductivity of the tissue. $p_{b}$ and $C_{b}$ are the density and specific heat of the blood; $w_{b}$ (mL/s/mL) is the blood perfusion rate; $O_{b}$ is the metabolic heat generation, $T_{a}$ is the supposedly constant arterial blood supplier temperature, and T is the breast temperature. To determine the relationship between depth and heat transfer during cooling, it is useful to define the depth of thermal penetration; unfortunately, this is not yet accurate due to the complex structure of the breast. However, a hypothesis can be made considering the hemispheric domain. Moreover, it appears that after cooling of the breast, the response time (the heat detected on the surface of the breast) increases with the depth of the tumor. We also observed that the diameter of the tumor has no significant effect on the response time for shallow tumors, and that the smaller the tumor, the longer the response time. During our experiment, we observed some principles to measure correct values, and also to make our thermal sensor more accurate [21]. One of the essential procedures that we found to be important was to maintain the room temperature between 18 and 22 °C, so that the total heat loss can be considered proportional to ($T_{s}$ − $T_{f}$), provided that ($T_{s}$ − $T_{f}$) is small. Thus, the boundary condition at the breast surface has been reduced as shown in Equation (2), with a surface conductance defined in Equation (3).

(2) $$-k{\frac{\partial T}{\partial t}|}_{skin}=h_{0}\left(T_{s}-T_{f}\right)$$

where *h*_0_ is called a constant of surface conductance which is composed of radiative and convective components.

(3) $$h_{0}=h_{conv}+h_{rad}$$

**Figure 8:**
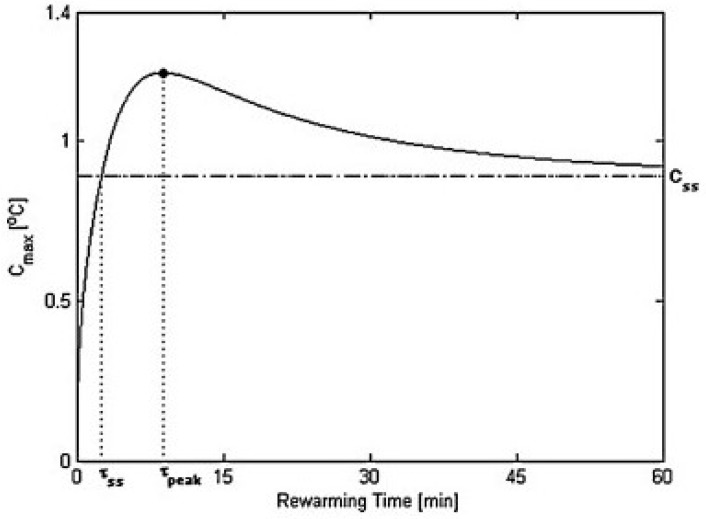
A phase of breast warming [17].

A typical maximum transient thermal contrast during warming for a breast with a 10 mm tumor at a depth of 5 mm after being cooled for 1 min was shown in this article. At the time of reading of the thermal graph processes by the computer, the amplitude of the transient peak and its corresponding time, as well as the response time, were extracted from the maximum transient thermal contrasts for tumors of different diameters located at different depths. This analysis shows the peak of the temperature generated on the surface of the breast with the tumor. As shown in the diagram in Figure 8, the area surrounding the tumor will produce more heat during the warming phase before falling back to a stable temperature.

**A genetic factor for breast cancer:** In the article by Cardoso [30], the analysis and summary of 12 short- and medium-term breast cancer clinical research projects suggested 12 areas of research to improve the detection and cure rates of breast cancer disease. Our point of interest (Developing Better Tools to Identify Genetically Predisposed Patients) was explored in the third search in this article, where they identified two significant genes (BRCA1 and BRCA2) in genetic testing for patients with a suggestive family history. It has emerged that confirmation of genetic predisposition can support the implementation of risk reduction strategies. Additionally, the use of new genetic testing tools, such as the high-risk hereditary breast cancer panel, should be accompanied by appropriate interpretation of results and variants so that they can be used in clinical decision-making. Recent studies show that poly (ADP-ribose) polymerase inhibitors (PARPs) may be useful in treating tumors harboring the BRCA1/2 mutations that develop breast cancer.

### 2.2. Economic Aspects of Breast Cancer

Breast cancer is the most common cancer in women, accounting for 23% of all newly diagnosed oncology cases. 85% of families in which one of the parents will have cancer are without the help of family, friends, non-profit organizations, or even debt. The management of patients with breast cancer has a significant impact on the national budget. It is, therefore, a public health priority to support effective prevention programs that could reduce cancer costs or provide therapeutic interventions that increase patients’ chances of survival, thus reducing the indirect costs of morbidity and mortality [31]. The reality of the cost burden is evidenced by numerous studies; for example, Smidova 2012 [28] showed that the productivity loss related to breast cancer in Poland amounted to 583.7 million EUR in 2010 and 699.7 million EUR in 2014. During this period, this amounts to 0.162–0.171% of the GDP. Public finance expenditure for social insurance benefits to breast cancer sufferers ranged from 50.2 million EUR (2010) to 56.6 million EUR (2014), or 0.72–0.79% of the total expenses for all diseases. Loss of opportunity in public finance revenue amounted to 173.9 million EUR in 2010 and 211.0 million EUR in 2014 [28].

**Figure 9:**
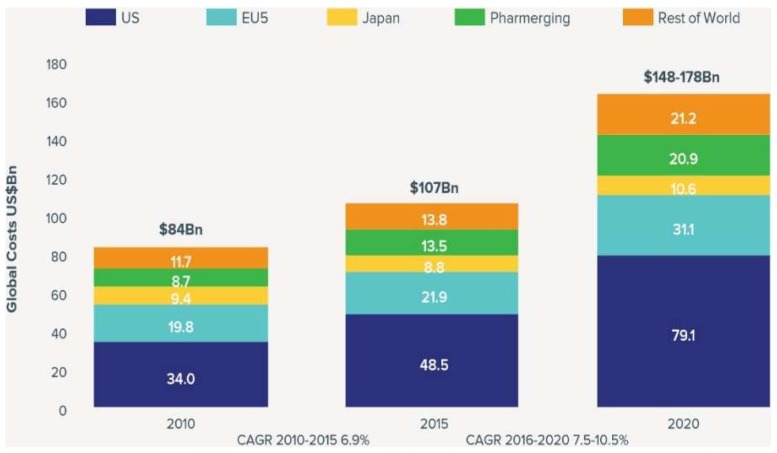
Global costs and growth of oncology, 2010–2020 [33].

Unar-Mungu’ıa et al., 2017 [24] estimated that 245 million USD in medical expenses and income lost due to breast cancer could be spared over the life of a cohort of Mexican women. Medical costs account for 80% of the economic burden; loss of income and opportunity costs for carers represent 15% and 5%, respectively [24]. Gustavsen et al., 2014 [32] assessed the utility of the Breast Cancer Index (BCI) cost for a US third-party payer. The use of the BCI is expected to reduce costs. In the newly diagnosed population, the net savings were $3803 per patient tested. In the five years following diagnosis, the BCI resulted in net cost savings of $1803 per patient tested. The prevalence of breast cancer treated increased from 7.9% to 20.4%. The total socio-economic costs incurred by breast cancer increased by approximately 40.7% compared to the United States. The cost of medical care in 2010 was 1.4 times higher (US $399.22 million) than in 2007 (US $278.71 million). Direct non-medical costs increased from US $50.69 million in 2007 to $75.83 million US dollars. Concerning the economic burden of breast cancer, the total indirect costs amounted to US $339.09 million in 2007 and increased by 37.3% to US $465.70 million in 2010. However, cost growth is not always linked to increasing prevalence, but also to the development of new healing methods. For example, standard chemotherapy for breast cancer in the Czech Republic costs around EUR 200. Biological treatment, which is suitable for one-fifth of patients, costs around EUR 30,000. The standard hormonal therapy is 200 EUR, while other hormonal preparations are worth 9000 EUR. The cost of radiotherapy [27,33] is between 1700 and 2600 EUR. Emerging gene or epigenetic therapies that directly influence the genetic information responsible for tumor growth will be even more expensive [26]. Oncology cost growth is expected to be between 7.5% and 10.5% per year by 2020, when global oncology costs will exceed $150 billion (Figure 9). Give these factors, the need to find a new method of detection or achieve even a small improvement in current processes is more than evident [21].

## 3. Discussion of Related Work

The study of the basis of breast cancer as it relates to modern techniques gives us a better view of the questions relating to the precise detection of tumors. Based on the articles discussed in the ”related work” section, we considered some features that we deem relevant enough to show a general difference between the Sentinel Breast Scan and the No Touch Breast Scan. It seems that most techniques today use a network of neurons to reduce the number of false positives (Table 1).

Through our research, we observed a considerable improvement in the detection of breast cancer in women under 50, with a sensitivity of ≥78% using No Touch software.

## 4. Proposed Model

For this work, the images were taken from the Research Data Base (DMR) containing frontal thermogram images, acquired using a FLIR SC-620 IR camera with a resolution of 640 × 480 pixels [24]. The dataset contains images of individuals aged between 29 and 85 years old. These images include breasts of different shapes and sizes, such as medium, wide, and asymmetric breasts.

The demographic data of the subjects are presented in Table 2, with:

- Total number of subjects (N) = 67.
- Total number of healthy/normal subjects (NH) = 43.
- Total number of sick/abnormal subjects (NS) = 24.

**Figure 10:**
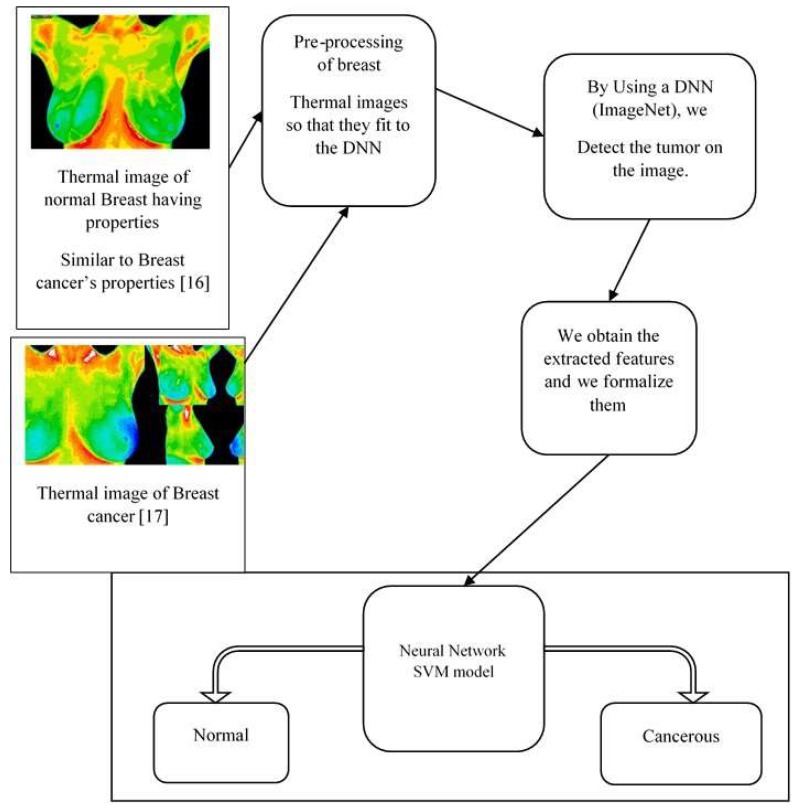
Illustration of our model [34].

Through the review of the articles discussed earlier, we noted the importance of image processing, which is currently performed well by a human being but which is not yet adequate when performed by artificial intelligence methods. This highlights the need for a Computer Assist Device (CAD) that will help us to better understand the thermal images captured by our different thermal imaging cameras [34]. In this context a CAD will be a deep neural network with an SVM model as a classifier, as shown in Figure 10 (assuming it is already trained) that will take the thermal images in, and as output classify the images as containing cancer or not. We should clarify that the deep learning module will output the probability of a breast’s thermal image being classified as sick (having cancer) or healthy (without cancer) [35]. If the confidence output (sick) by our DNN is greater than 0.5 and less than 0.6, it will be up to our Super Machine Vector classification to distinguish the state of the patient’s breast based on the feature matrix of the breast thermal image.

Our proposed architecture (Figure 10) can be likened to a flow chart where each module represents a specific component.

### 4.1. Why a New Model?

In Section 2, we outlined previous and new techniques used for the detection and prevention of breast cancer. We aim to contribute to this challenging task with our work. 

The model that we present in this paper takes advantage of two main factors:

- It uses a deep neural network (a pre-trained Inception V3 model [36,37]) which is modified at the last fully connected layer in such a way as to obtain a powerful binary classification (sick breast or healthy breast).
- A second well known classifier (SVM) is coupled to that, and is involved only in the case of an uncertainty in the output of the DNN.

### 4.2. Pre-Processing of Breast Thermal Images:

We can subdivide this task as follows:

- Pre-treatment of the breast thermal images: We chose to use a well-known dataset [7]. The thermal images of this dataset were obtained by following a dynamic protocol, which consists of taking picture after the cooling of breasts by air stream. During the process of returning the patient’s body to thermal equilibrium with the environment, the author of the dataset obtained 20 sequential images with intervals of 15 s between them. The images in their original input format (640 × 480) are very large for our DNN, so it is important to crop them to remove unwanted areas.
- Obtaining the Region of Interest: From each grey scale image, the Region of Interest (ROI) was extracted. Each ROI image is converted into a matrix of characteristics that will be processed, and the areas most likely to have cancer will be transferred to the input of the next component.

Thus, pre-processing involves grayscale RGB conversion, and image culture to remove unwanted regions such as the neck region, arms, and sub-membrane folding of the region.

### 4.3. Image Classification Framework

Considering the concept of transfer learning, we used a pre-trained Inception V3 model [37], which is powerful enough for feature extraction. Our DNN can be described as [25,38], which means there is a layer of 10 neurons, where each is connected to 20 neurons in the next layer, and similarly each is connected to 10 neurons in the third layer. In addition, we retrained the final classified layer so that it could determine cancer versus no cancer with considerable confidence (>0.6). If the final layer output had a confidence of <0.6 and ≥0.5, we submitted the matrix of features to our support vector machine (SVM) for output prediction. During the training of our model, we set the learning rate at 0.0001, the epochs to 15, and the steps to 4000 (all these numbers were obtained through several experiments). Furthermore, our training was done using a sample of 64 breasts, including 32 that were healthy and 32 with some abnormality, where each breast had 20 sequential images. This resulted in 1062 images (after the pre-processing phase) used to train and test our network with respective reparations of 0.8 and 0.2 as shown on Table 3. To validate the performed tests, we used 12 breasts that were totally new to our model.

**Figure 11:**
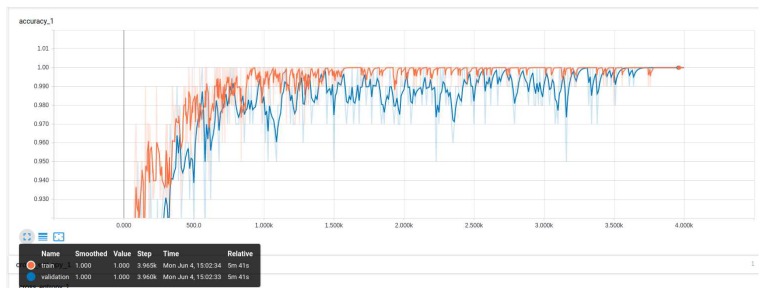
Illustration of the increase in the accuracy over the number of iterations. The training and the validation become stable after 3900 training steps.

**Figure 12:**
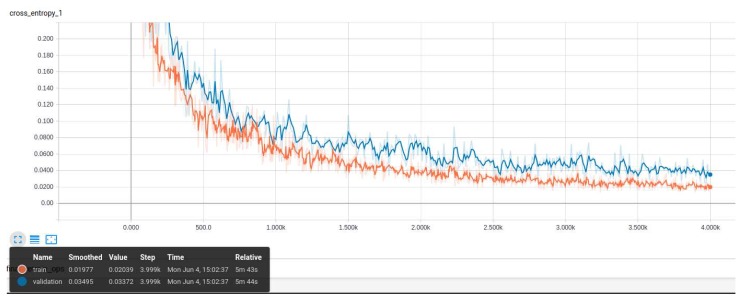
The entropy decreases during the training of our model, which can be considered a positive sign (the ambiguity reduces in our model).

InceptionV3 Model: During the training of our InceptionV3 model (Figure 11 and Figure 12), we observed increased accuracy and the reduction of the entropy of our model after 3900 steps. Above this number of steps, the model will be over-fit and this will cause a decrease in the accuracy.

**Figure 13:**
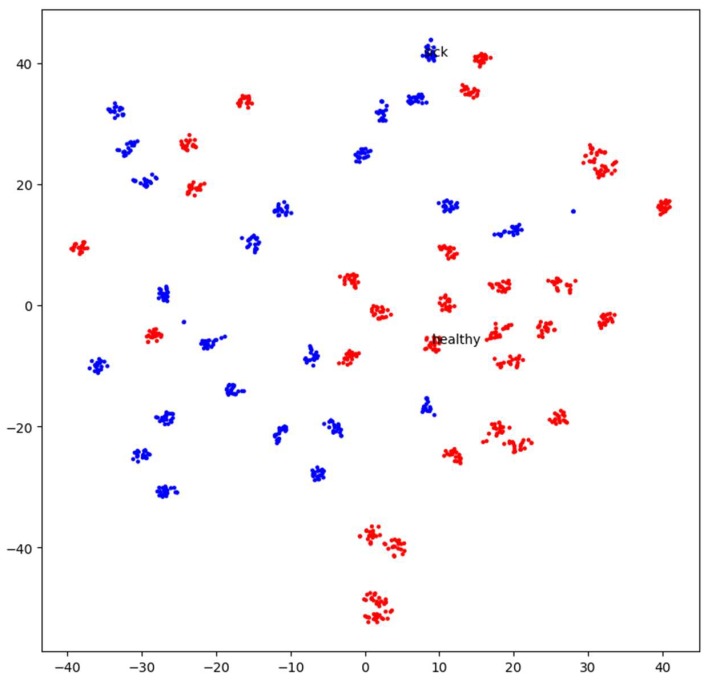
The spatial distribution of our (retrained InceptionV3) model extracted feature representation shows how well features are grouped. In Red, we see elements classified as Healthy (For Healthy Breast), and In Blue, we see features organized as Sick (For Sick Breast or Breast with Cancer).

**Figure 14:**
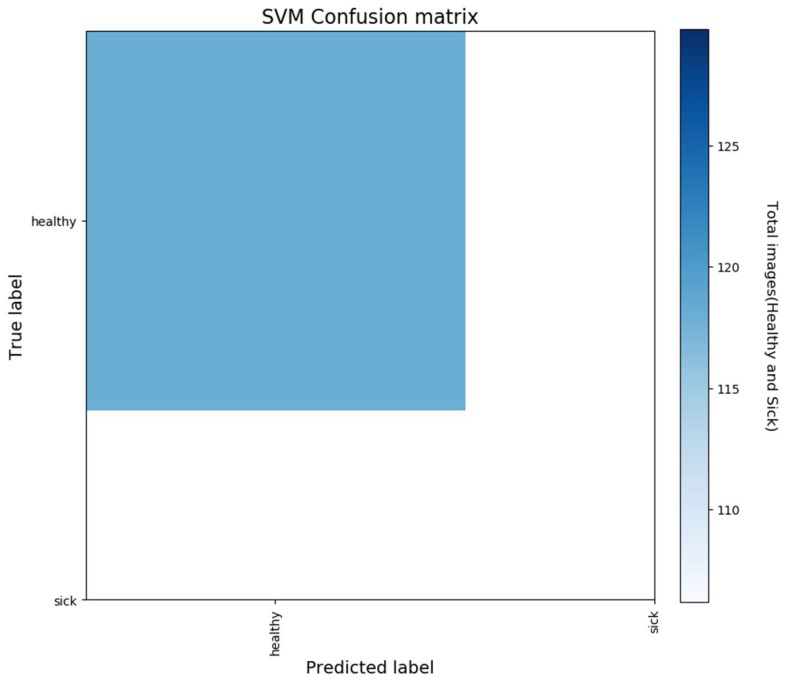
Confusion Matrix of our LinearSVC (SVM) shows how confident our classifier is for each prediction.

**Figure 15:**
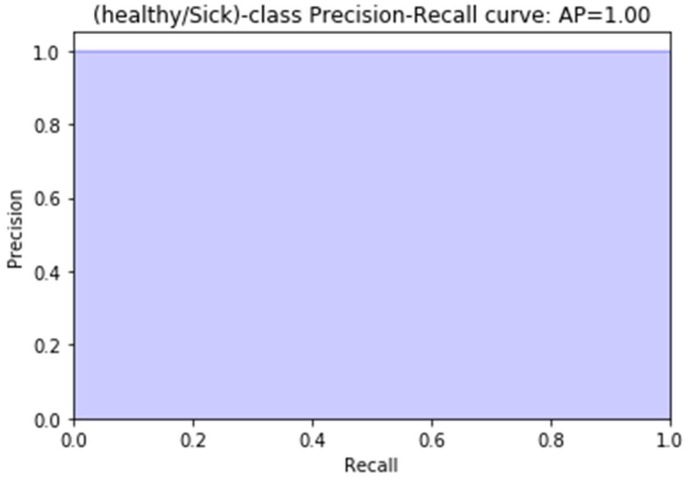
Precision-Recall of our LinearSVC result is a useful measure of the success of our prediction. In information retrieval, precision is a measure of result relevancy, while recall is a measure of how many truly relevant results are returned.

**Figure 16:**
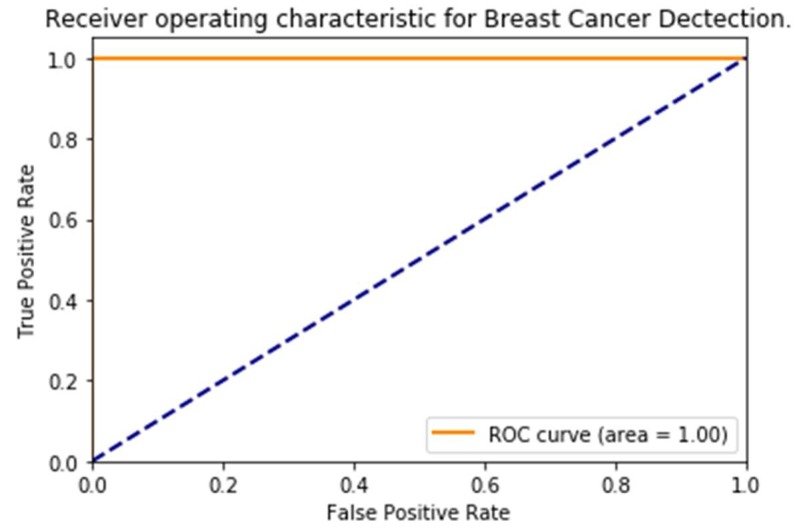
A receiver operating characteristic (ROC) curves of our prediction, where the top left corner of the plot can be interpreted as “ideal” point (a false positive rate of zero, and a true positive rate of one).

Super Vector Machine (SVM) Model: As shown in Figure 13, our extracted features representation shows that our features are not entirely classified. We can distinguish two main group Healthy and Sick. Our goal here is to perform an additional classification on top of it so that we can have better accuracy for our classification. We chose for this purpose, an inbuilt variation of an SVM which is a Linear Super Vector Classification (LinearSVC). As mention in [39], it has more flexibility, supports both dense and sparse input, and it is suitable for the multiclass classification. The performance of our LinearSVC is illustrated in Figure 14, Figure 15 and Figure 16.

**Figure 17:**
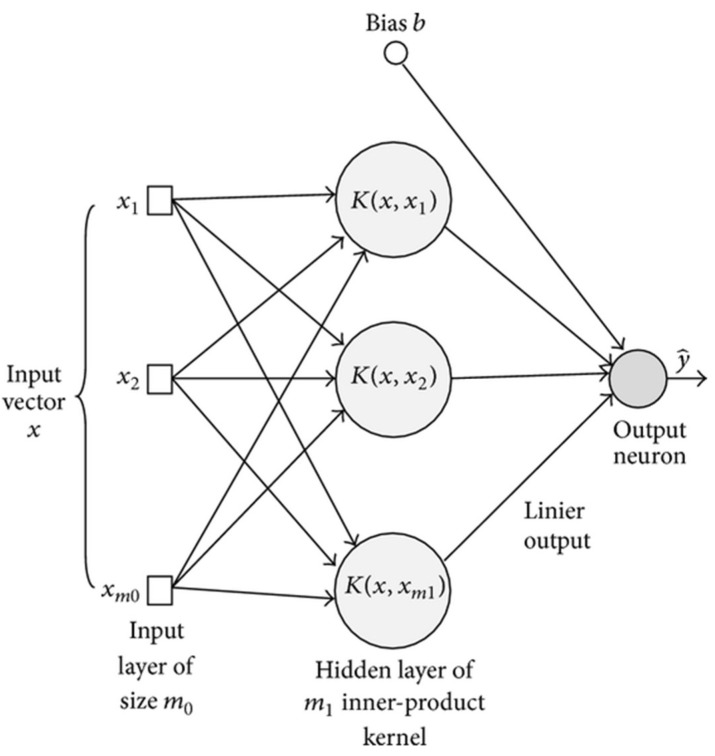
Our SVM architecture. The input Vector our context is filled by the features extracted at the feature layer of our Inception V3.

As shown in Figure 17, we are using a simple linear SVM architecture with a first layer acting as input layer fill by the features obtained from our InceptionV3, our hidden layer which contains several neurons and performs an inner-product [40].

### 4.4. Results

**Figure 18:**
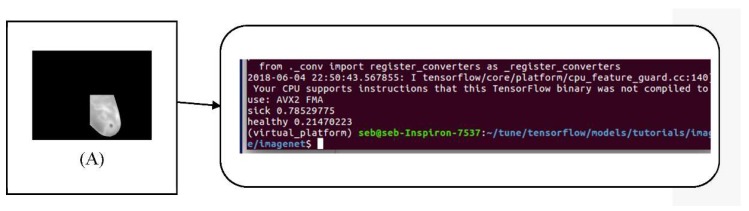
Given image (**A**) as input, our model classifies the image as “sick” with a confidence of 0.78.

**Figure 19:**
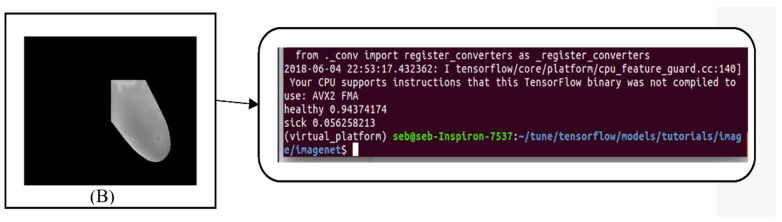
Given image (**B**) as input, our model classifies the image as “healthy” with a confidence of 0.94.

After the training of our model, we performed the validation test by taking the breast images from the dataset of 12 new breasts (as mentioned previously). Keeping in mind that for each breast we have 20 sequential images, we submitted 480 images for the validation test organize according to Table 4. Figure 18 and Figure 19 show the predictions of our model.

## 5. Conclusions

During the review of the literature, it became apparent that work in the area of breast cancer detection from a computer scientist point of view could be a valuable contribution to the field. With this in mind, we presented the techniques most commonly used to detect breast cancer, and their strengths and weaknesses. One technique in particular appeared to have a promising future, because of its non-immersive property and the significant amount of data that needs to be processed with more efficient techniques. Infrared imaging coupled with an agent previously administered to a patient can lead to a very accurate tumor detector. In a future study, we will use a thermal sensitivity camera of 0.5, and propose a model of the breast which will be able to help us to realize an even more precise diagnosis.

## Figures and Tables

**Table 1:** Difference between the Sentinel Breast Scan and No Touch Breast Scan.

Feature	Non-Contact Breast Scan	Sentinel Breast Scan
IR Camera resolution given in pixels	640 × 512	320 × 240
Temperature sensitivity in °C	0.05	0.08
Number of IR cameras	2	1
Transient IR	Yes	Yes
Wavelength range	3.5–10.5	7–12
Cooling time in min	5–6	3–6
Analysis time in min	Immediate	4–5
Cooling method	Cold Air	Cold Air
Deep Neural network (DNN)	Yes	Yes
Age < 50 sensitivity with DNN [32]	78%	67%
Age < 50 sensitivity without DNN [32]	89%	78%

**Table 2:** Summary of subjects.

Age Range	Total Number of Sick	Total Number of Healthy
29–50	11	07
51–70	12	31
71–85	01	05

**Table 3:** Dataset repartition use for training.

	Training	Testing	Total
Healthy	481	121	602
Sick	368	92	460
Total	849	213	1062

**Table 4:** Dataset reparation use for validation test.

	Testing
Healthy	300
Sick	180
Total	480
